# Hypertension in Dialysis Patients: Diagnostic Approaches and Evaluation of Epidemiology

**DOI:** 10.3390/diagnostics12122961

**Published:** 2022-11-26

**Authors:** Panagiotis I. Georgianos, Vasilios Vaios, Vasiliki Sgouropoulou, Theodoros Eleftheriadis, Dimitrios G. Tsalikakis, Vassilios Liakopoulos

**Affiliations:** 1 Section of Nephrology and Hypertension, 1st Department of Medicine, AHEPA Hospital, Aristotle University of Thessaloniki, 54124 Thessaloniki, Greece; 2 Department of Nephrology, School of Medicine, University of Thessaly, 41222 Larissa, Greece; 3 Department of Electrical and Computer Engineering, University of Western Macedonia, 50100 Kozani, Greece

**Keywords:** hypertension, dialysis, diagnosis, epidemiology, home BP monitoring, ambulatory BP monitoring

## Abstract

Whereas hypertension is an established cardiovascular risk factor in the general population, the contribution of increased blood pressure (BP) to the huge burden of cardiovascular morbidity and mortality in patients receiving dialysis continues to be debated. In a large part, this controversy is attributable to particular difficulties in the accurate diagnosis of hypertension. The reverse epidemiology of hypertension in dialysis patients is based on evidence from large cohort studies showing that routine predialysis or postdialysis BP measurements exhibit a U-shaped or J-shaped association with cardiovascular or all-cause mortality. However, substantial evidence supports the notion that home or ambulatory BP measurements are superior to dialysis-unit BP recordings in diagnosing hypertension, in detecting evidence of target-organ damage and in prognosticating the all-cause death risk. In the first part of this article, we explore the accuracy of different methods of BP measurement in diagnosing hypertension among patients on dialysis. In the second part, we describe how the epidemiology of hypertension is modified when the assessment of BP is based on dialysis-unit versus home or ambulatory recordings.

## 1. Introduction

Hypertension among patients with end-stage kidney disease (ESKD) receiving either long-term hemodialysis or peritoneal dialysis (PD) is highly prevalent and often remains poorly controlled [1,2]. Two meta-analyses of randomized controlled trials have demonstrated that the treatment of dialysis patients with blood pressure (BP)-lowering medications is effective in reducing the incidence of adverse cardiovascular events, the leading cause of mortality in this high-risk population [3,4]. However, there is currently no consensus on whether increased BP is an independent cardiovascular risk factor or on the optimal BP targets [5]. In a large part, this controversy is attributable to particular difficulties in diagnosing hypertension in these patients accurately.

In the first part of this article, we explore the accuracy of various BP monitoring techniques in diagnosing hypertension among patients on dialysis. In the second part, we describe how the epidemiology of hypertension is modified when different methods are used in the assessment of BP.

## 2. Literature Review

A literature search of the MedLine/PubMed database was performed from inception until September 2022 with the aim to identify studies that explored the diagnostic accuracy and predictive value of different BP monitoring techniques in ESKD patients on dialysis. Search terms used were “hypertension”, “hemodialysis”, “peritoneal dialysis”, “end-stage kidney disease”, “home BP monitoring” and “ambulatory BP monitoring”. Reference lists of prior reviews in this area were also searched for additional studies and information. All types of studies, such as diagnostic-test studies, cross-sectional analyses, prospective observational studies, randomized controlled trials and meta-analyses, were eligible for this article if they reported relevant data with respect to the diagnosis and epidemiology of hypertension. Studies that were conducted in the general hypertensive population or in patients with earlier stages of kidney disease were excluded.

## 3. Diagnosis of Hypertension

### 3.1. Peridialytic BP Measurements

Peridialytic BP measurements are the recordings obtained by the dialysis-unit personnel at the beginning and at the end of each hemodialysis session [1,2]. These measurements remain the most commonly applied approach for the diagnosis of hypertension, mostly because predialysis and postdialysis BP recordings are obtained in a systematic manner and are easily available to the nephrologist at the bedside. However, a 2006 meta-analysis of 18 diagnostic-test studies showed that routine peridialytic BP recordings provide an inaccurate reflection of the actual BP load, assessed using the reference-standard technique of 44-hour ambulatory BP monitoring (ABPM) [6]. Peridialytic BP continues to be insufficient for diagnosing hypertension, even when a higher number of recordings are averaged over six consecutive hemodialysis treatments [7]. On this basis, excessive variability in BP either from pre- to postdialysis or from one hemodialysis session to the next is not the only factor that disturbs the diagnostic accuracy of these measurements [8]. Another potential source of bias could be the lack of a standardized methodology in BP measurement. However, even when these measurements are obtained in a standardized manner (i.e., with a validated BP monitor after a prespecified 5 min rest period) and averaged over 2 weeks, peridialytic BP recordings are still inaccurate in diagnosing hypertension [9]. Furthermore, routine and standardized peridialytic BP exhibits a similarly weak correlation with the severity of hypertension-related target-organ damage, such as echocardiographically documented left ventricular (LV) hypertrophy [10]. Most importantly, large cohort studies have consistently revealed a reverse U-shaped or J-shaped relation of peridialytic BP with the risk of cardiovascular morbidity and mortality (Table 1) [11,12,13,14,15].

### 3.2. Intradialytic BP Measurements

One approach to improve the diagnostic performance of peridialytic BP measurements is the concomitant assessment of intradialytic BP. These measurements are obtained during the hemodialysis procedure, typically every 30 min, using an automatic cuff attached to the hemodialysis machine [1,2]. In daily clinical practice, these measurements are essential for monitoring the hemodynamic stability of the patients during the intradialytic period. An earlier diagnostic-test study showed that when the average intradialytic BP is considered jointly with peridialytic BP recordings, there is improvement in the diagnostic accuracy of BP measurement as compared to the use of predialysis and postdialysis BP measurements alone [24]. This observation is consistent with the results of a recent study showing that the sensitivity and specificity for the diagnosis of an average 44-hour ambulatory systolic BP ≥ 130 mmHg were low for predialysis (86.5%/38.6%) and postdialysis BP (63.1%/73.3%), but better for the average intradialytic BP (77.3%/76.2%) and the combination of intradialytic plus peridialytic BP (76.6%/72.3%) [25].

Since the calculation of an average is time-consuming and impractical, consideration of the median intradialytic BP from the measurements obtained during a single mid-week hemodialysis session also appears to be an appropriate method to screen for hypertension at the bedside. A mid-week median cut-off systolic BP of 140 mmHg provides 80% sensitivity and 80% specificity for the diagnosis of systolic hypertension. Similarly, a mid-week median intradialytic diastolic BP of 80 mmHg offers 75% sensitivity and 75% specificity in the detection of diastolic hypertension [24]. Furthermore, in the Dry Weight Reduction in Hypertensive Hemodialysis Patients (DRIP) trial, median mid-week intradialytic BP was volume-sensitive and could track with accuracy changes in 44-hour ambulatory BP provoked by the gentle and gradual reduction in dry weight over the course of the trial [26]. It has to be noted, however, that the use of mid-week median intradialytic BP for the diagnosis of hypertension is a method of last resort. As discussed in detail below, BP measurements taken outside of dialysis are more reliable for the assessment and management of hypertension in the long-term.

### 3.3. Home BP Measurements

Home BP monitoring (HBPM) is an established technique that is recommended by international guidelines for the assessment and management of hypertension in the general population or in patients with predialysis chronic kidney disease [27]. However, the adoption of this technique in hemodialysis patients has been low, despite the fact that diagnostic-test studies have demonstrated that HBPM can facilitate the diagnosis of hypertension. Using 44-hour ABPM as the reference-standard method, Agarwal at al. [16] showed that the area under the receiver operating characteristics (ROC) curve was similar for home and standardized peridialytic BP but lower for routine predialysis and postdialysis BP. One-week averaged home systolic BP of ≥ 150 mmHg provided 80% sensitivity and 84.1% specificity in diagnosing ambulatory hypertension [16]. By contrast, there was no cut-point for routine or standardized peridialytic BP to ensure the detection of hypertension with high precision.

The technique of HBPM has been more widely adopted in the assessment of hypertension in patients receiving long-term PD, possibly because PD is a home-based modality for renal replacement therapy. Among 81 stable PD patients, a recent study showed that self-measured home BP for 1 week had at least similar or even better accuracy than standardized dialysis-unit BP in diagnosing hypertension, as confirmed by the reference-standard method of 24-hour ABPM [18]. The area under the ROC curve for the detection of an average ambulatory daytime systolic BP of ≥ 135 mmHg did not differ between standardized dialysis-unit (area under curve: 0.859; 95% confidence interval (CI): 0.776–0.941) and 1-week averaged home systolic BP (area under curve: 0.895; 95% CI: 0.815–0.976). In Bland–Altman plots, standardized dialysis-unit systolic BP and 1-week averaged home systolic BP overestimated daytime ambulatory systolic BP by 5.02 mmHg and 4.23 mmHg on average, respectively [18].

Apart from its superior diagnostic performance, additional advantages of the technique of HBPM exist and merit attention. In DRIP, home BP was more sensitive than peridialytic BP in detecting the reduction in 44-hour ambulatory BP provoked by the probing of dry weight over 8 weeks of follow-up [17]. Contrary to the poor reproducibility of predialysis and postdialysis BP, home BP had even greater test–retest reliability than 44-hour ambulatory BP [17]. Home BP is superior even to standardized peridialytic BP in detecting the presence of echocardiographically documented LV hypertrophy [10]. Unlike the reverse relation between peridialytic BP and all-cause mortality, increasing levels of home BP provide a clear and direct mortality risk signal [19,20].

Substantial evidence also supports the notion that HBPM is a useful tool to guide therapeutic decisions and improve the management of hypertension [5]. The feasibility of and adherence to a home-BP-guided antihypertensive strategy was investigated in the Blood Pressure Lowering in Dialysis (BOLD) trial [28]. Home BP (in the intervention group) and predialysis BP (in the control group) were evaluated every 2 weeks to guide the adjustment in dry weight and intensity of BP-lowering treatment. Of the 70 patients who were approached, 50 patients (71%) agreed to take part in the trial [28]. Of the 50 patients enrolled in the trial, 49 patients successfully completed the protocol procedures until the end of follow-up. In the intervention group, adherence to obtaining and reporting home BP was 97.4%; this high adherence rate remained consistent over a prospective period of 4 months [28]. In an open-label trial, 65 hemodialysis patients with uncontrolled hypertension were randomized to home-BP-guided versus predialysis-BP-guided treatment of hypertension over 6 months [21]. As compared to usual care, the utilization of HBPM in the intervention group provoked a statistically significant reduction of ~9/7 mmHg in interdialytic ambulatory BP [21]. The technique of HBPM was successfully utilized in the Hypertension in Hemodialysis treated with Atenolol or Lisinopril (HDPAL) trial [29]. In HDPAL, monthly measured home systolic BP was targeted to levels <140 mmHg in 200 hypertensive hemodialysis patients using a combined therapeutic strategy that included dietary sodium restriction, the probing of dry weight and intensification of BP-lowering medications. Home-BP-guided therapeutic decisions were accompanied by a clinically meaningful improvement in the control of 44-hour ambulatory BP over 12 months of follow-up [29].

The chronobiology of BP should be taken into consideration in the design of a sufficient scheme for home BP measurements. Home systolic BP is characterized by a steady increase at a rate of 0.40 ± 0.25 mmHg/hour over the interdialytic interval. This rising pattern reaches a plateau after approximately 48 h [30]. Therefore, the time elapsed from hemodialysis is important, because home BP measurements taken immediately postdialysis or shortly before the hemodialysis session may underestimate or overestimate the actual BP load. In a diagnostic-test study, home BP recordings that were taken at each third of the interdialytic period provided the highest accuracy in the diagnosis of hypertension [30]. Furthermore, the HBPM schemes should be accordant with what current international guidelines define as the minimum dataset of BP measurements for diagnosis of hypertension, detection of hypertension-related target-organ damage and prognostication of all-cause death risk. According to guidelines, HBPM should cover at least 3 and preferably 7 consecutive days [31]. Therefore, a practical approach is to ask the patients to perform HBPM twice daily (on waking and at bedtime) for 4 days after the completion of the mid-week hemodialysis session. This abbreviated protocol was implemented in HDPAL and provided sufficient data for the guidance of antihypertensive therapy over the course of this trial [29].

### 3.4. Ambulatory BP Measurements

Interdialytic ABPM represents the “reference-standard” technique for diagnosing hypertension [1,2]. This method is valid [32], reproducible [23], and provides higher accuracy as compared to peridialytic BP measurements in the detection of LV hypertrophy [10] or in the prediction of the long-term risk for cardiovascular morbidity and mortality [19,20,33]. To ensure the highest diagnostic precision, ABPM should cover the entire interdialytic period (44 h). In this technique, BP is typically recorded three times per hour during the daytime period and two times per hour during the nighttime period [22]. The availability of more measurements enables the more detailed evaluation of the chronobiology of BP. Ambulatory systolic BP increases in a steady manner during the interdialytic interval, but the rate of increase is slower than that of home systolic BP (0.30 ± 0.36 mmHg/hour) [34]. The strength of ABPM is that this technique enables the measurement of BP during periods of activity, whereas home BP is typically recorded during periods of resting. Another unique advantage of this technique is that BP can be recorded during the period of sleep, facilitating the diagnosis of nocturnal hypertension and non-dipper/reverse-dipper BP patterns. These phenotypes are very common in patients on dialysis, and their presence goes hand in hand with greater severity of target-organ damage and with enhanced risk of mortality [35,36,37]. However, some weaknesses, such as the limited availability and costs of equipment, need for training and patient discomfort, limit the wide use of ABPM in daily clinical practice.

## 4. Epidemiology

### 4.1. Prevalence, Treatment and Control of Hypertension

Studies exploring the epidemiology of hypertension in patients on dialysis have used different definitions and provided considerably variable results with respect to the prevalence and control of BP [38,39,40]. The epidemiology of hypertension varies across studies, depending on the technique of BP measurement [1,2]. For example, in a cross-sectional study enrolling 116 hemodialysis patients from Northern Greece, the prevalence of hypertension was 88.8% using home, 86.2% using predialysis and 91.4% using postdialysis BP measurements [41]. Overall, 96 out of 116 patients were receiving therapy with an average of 2.0 antihypertensive drugs daily. The control rates of hypertension differed according to the method of BP measurement: 32.6% of drug-treated patients were controlled using home, 50.5% using predialysis and 45.3% using postdialysis BP recordings [41]. The agreement between predialysis BP recordings and HBPM in the diagnosis of uncontrolled hypertension was poor (k-statistic: 0.430). The agreement between postdialysis BP measurements and HBPM was even worse (k-statistic: 0.299) [41].

A more accurate description of the epidemiology of hypertension was provided by two larger studies that incorporated the reference-standard method of ABPM [42,43]. The first study included 369 hemodialysis patients and showed that the prevalence of hypertension (defined as 44 h ambulatory BP ≥ 135/85 mmHg or antihypertensive drug use) was 82% [42]. Despite the fact that 89% of hypertensives were receiving treatment, adequate control of 44 h ambulatory BP was achieved in 38% [42]. In a subsequent study involving 396 hemodialysis patients from the European Cardiovascular and Renal Medicine (EURECA-m) Registry of the ERA-EDTA, hypertension (defined as 48 h ambulatory BP ≥ 130/80 mmHg or current use of BP-lowering medications) was prevalent in 84.3% of patients [43]. Although 86.8% of hypertensives were receiving pharmacological treatment, only 28.7% had their ambulatory BP adequately controlled [43]. Taken together, although these two studies utilized different monitoring schemes and BP thresholds to define hypertension, both of them showed that the burden of hypertension in the hemodialysis population is high.

Some earlier studies that were based on conventional dialysis-unit BP recordings suggested that hypertension control in patients receiving long-term PD is superior compared to those on hemodialysis. For example, among 1202 patients enrolled in the 1995 Peritoneal Dialysis Core Indicators Study, only 29% had an office systolic BP > 150 mmHg and 18% had a diastolic BP > 90 mmHg [44]. However, these earlier estimates are not accordant with the results of a recent analysis of 140 stable ESKD patients from four PD centers in Northern Greece showing a high burden of hypertension in the PD population as well [45]. In this study, the prevalence of hypertension was 92.9% using a diagnostic threshold of 140/90 mmHg for standardized office BP recordings, whereas the prevalence rate of hypertension was 95% when a threshold of 130/80 mmHg for 24-hour ambulatory BP was applied. The vast majority of patients (92.1%) were taking antihypertensive medications. However, only 52.3% and 38.3% of hypertensives were adequately controlled using office BP measurements and ABPM, respectively [45]. Studies using a case–control design also showed that the mean levels and trajectories of ambulatory BP did not significantly differ when patients on PD were compared with age-, sex- and dialysis-vintage-matched controls on thrice-weekly hemodialysis [46].

### 4.2. Prognostic Association of BP with Mortality

The risk association of BP with cardiovascular morbidity and all-cause mortality among patients on dialysis represents an area of controversy [12]. In the general population, there is a direct and linear association between BP and outcome [47,48]. In contrast, large observational studies and a recent meta-analysis revealed a U-shaped or J-shaped association of predialysis or postdialysis BP with all-cause death risk [11,12,13,14,15]. The phenomenon of lower BP being linked with an increased mortality has been described as “reverse epidemiology” of hypertension. This paradoxical phenomenon has raised questions on whether hypertension in the dialysis population is truly an independent risk factor that should be treated and controlled [12].

Recent studies have shown that peridialytic BP should be interpreted within the context of hypervolemia and not as an isolated risk factor. In an analysis from the international Monitoring Dialysis Outcome Initiative (MONDO) database, the volume status of 8883 hemodialysis patients was assessed using bioimpedance spectroscopy [49]. All-cause mortality was prospectively assessed over 1 year. In patients with hypervolemia, low predialysis systolic BP <110 mmHg was associated with an increased risk of mortality (hazard ratio (HR): 1.52; 95% CI: 1.06–2.17). An increased mortality risk was also detected in patients who were volume-depleted and had a predialysis systolic BP within the normotensive range (i.e., <140 mmHg). By contrast, among euvolemic patients, low predialysis systolic BP <110 mmHg was associated with improved survival (HR: 0.46; 95% CI: 0.23–0.91) [49]. Other confounding parameters with an opposing impact on BP, such as the level of illness or the history of severe heart failure, may also limit the ability of predialysis and postdialysis BP to provide a direct mortality risk signal [12].

Two separate prospective observational studies showed that the BP measurement technique is another factor that modifies the prognostic association of BP with all-cause mortality. The first study enrolled 150 hemodialysis patients who underwent a baseline assessment of BP with four different techniques: self-measured home BP for 1 week, 44 h ABPM and routine and standardized peridialytic BP measurements averaged over six consecutive hemodialysis sessions [20]. During a prospective period of 24 months, 46 patients died. Each 1 standard deviation (SD)-higher home systolic BP was associated with a 35% greater risk of all-cause mortality (HR: 1.35; 95% CI: 0.99–1.84). Similarly, each 1 SD increase in 44 h ambulatory systolic BP was associated with a 46% greater risk of all-cause mortality (HR: 1.46; 95% CI: 1.09–1.94) [20]. In sharp contrast, both routine and standardized peridialytic BP recordings had no significant association with the risk of all-cause mortality.

The second study included a larger sample size of 326 hemodialysis patients who underwent a similar assessment of BP at baseline. The duration of follow-up was longer also (median follow-up: 32 months) [19]. In multivariable-adjusted Cox-regression models, compared with the referent quartile 1, the HR for all-cause mortality was 2.15 (95% CI: 1.13–4.11) in quartile 2, 1.7 (95% CI: 0.88–3.29) in quartile 3 and 1.44 (95% CI: 0.72–2.9) in quartile 4 of home systolic BP. In a similar fashion, using the quartile 1 of 44 h ambulatory systolic BP as a reference category, the HR for all-cause mortality was 2.51 (95% CI: 1.27–4.95) in quartile 2, 3.43 (95% CI: 1.73–6.79) in quartile 3 and 2.62 (95% CI: 1.33–5.17) in quartile 4 [19]. When the method of restricted cubic splines was used, a W-shaped risk association between both home and ambulatory systolic BP and all-cause mortality was observed. Home systolic BP within the range of 120 to 130 mmHg and 44 h ambulatory systolic BP within the range of 110 to 120 mmHg were associated with the greatest survival [19].

The notion that ambulatory BP provides superior prognostic information relative to office BP is further supported by a prospective cohort study of 108 stable PD patients [50]. At baseline, standardized office BP measurements and 24-hour ABPM were performed. During 16 months of prospective follow-up, increasing levels of 24-hour ambulatory systolic BP were associated with a progressively higher risk for the composite outcome of non-fatal myocardial infarction, non-fatal stroke or death from any cause (multivariate-adjusted HR: 1.098, 1.004 and 2.449 for quartiles 2, 3 and 4 of 24-hour ambulatory systolic BP, respectively) [50]. However, no such dose–response relationship was seen between increasing quartiles of the office systolic BP and the risk for the composite outcome.

The superior prognostic significance of home or ambulatory measurements over dialysis-unit BP cannot be attributed to the higher number of BP recordings that these techniques provide. Observational studies showed that both home and ambulatory BP retain their ability to prognosticate the all-cause death risk, even when the average of a small number of randomly selected BP recordings is used as a risk predictor [51,52,53,54]. Therefore, it is the setting of measurement, not the number of BP recordings, the factor that modifies the risk association between BP and all-cause mortality.

The reverse epidemiology of hypertension demonstrated by observational data is not accordant with the results of two earlier meta-analyses of randomized controlled trials showing that deliberate lowering of BP with the use of antihypertensive therapy offers cardiovascular protection in patients on dialysis. The first meta-analysis included eight trials involving a total of 1679 dialysis patients. Relative to placebo or no therapy, the use of antihypertensive medications was associated with 29% reduced risk for cardiovascular events (risk ratio (RR): 0.71; 95% CI: 0.55–0.92), 29% lower risk for cardiovascular death (RR: 0.71; 95% CI: 0.50–0.99) and 20% lower risk for all-cause death (RR: 0.80; 95% CI: 0.66–0.96) [4]. The second meta-analysis included 5 trials with 1212 participants. As compared with placebo, antihypertensive therapy was associated with a reduction of 31% in the risk for adverse cardiovascular events and all-cause mortality (HR: 0.69; 95% CI: 0.56–0.84) [3]. The cardiovascular benefit of antihypertensive therapy was higher when trials with hypertensive patients were combined separately from trials that included only normotensive patients at baseline. Taken together, these meta-analytic data show a clear cardioprotective benefit of antihypertensive therapy in dialysis patients, particularly in those with hypertension [3].

## 5. Conclusions

In conclusion, although the present work provides mainly a qualitative evaluation of diagnostic accuracy and predictive value of different BP monitoring techniques in the dialysis population, a growing body of evidence supports that home and ambulatory BP measurements are superior to routine and standardized dialysis-unit BP recordings in diagnosing hypertension, in assessing the presence of target-organ damage and in prognosticating the risk of all-cause mortality. Undoubtedly, 44 h ABPM is the “gold-standard” technique that provides the highest accuracy and precision, but some weaknesses (i.e., the low availability and cost of equipment as well as patient discomfort) limit the adoption of this method in daily clinical practice. A more feasible approach to improve the assessment and long-term management of hypertension is the broader use of HBPM. Large randomized controlled trials are now needed to demonstrate the superiority of home-BP-guided over predialysis-BP-guided antihypertensive therapy in causing regression of end-organ damage and in improving “hard” cardiovascular outcomes in this high-risk patient population.

## Figures and Tables

**Table 1:** Techniques for BP measurement.

Technique	Description	Advantages	Disadvantages
Routine peridialytic BP	BP recordings taken shortly before or after hemodialysis without a standardized methodology in BP measurement.	Systematically recorded and easily available at the bedside. No need for training.	Poor diagnostic accuracy [6]. Poor reproducibility [8]. Weak correlation with target-organ damage [10]. U-shaped or J-shaped association with all-cause mortality [12].
Standardized peridialytic BP	BP recordings before or after hemodialysis taken under standardized conditions (i.e., after a 5 min seated rest and with a validated oscillometric device).	Easily available. No need for training.	Poor diagnostic accuracy [16]. Poor reproducibility [17]. Weak correlation with target-organ damage [10]. Poor prognostic value [12].
Home BP	Self-measured BP recordings taken at home with a validated oscillometric device twice daily for at least 3 and preferably 7 consecutive days.	Greater diagnostic accuracy than standardized peridialytic BP [16,18]. Better reproducibility [17]. Closer association with target-organ damage [10]. Prognostically informative [12,19,20]. Useful for long-term guidance of antihypertensive therapy [21].	No assessment of BP during nighttime. Assessment of BP only during periods of resting. Need for training.
Ambulatory BP	Automated BP recordings, typically 3 times per hour during the daytime period and 2 times per hour during the nighttime period over an entire interdialytic interval (44 h).	The reference-standard method that enables the assessment of BP during periods of activity and during periods of sleep [22]. Better reproducibility [23]. Closer association with target-organ damage [10]. Prognostically informative [12,19,20].	Limited availability in daily clinical practice. Patient discomfort to perform the measurement repeatedly in the long term. Need for training.

Abbreviations: BP = blood pressure.

## Data Availability

Not applicable.
